# Mosquito Innate Immunity

**DOI:** 10.3390/insects9030095

**Published:** 2018-08-08

**Authors:** Ankit Kumar, Priyanshu Srivastava, PDNN Sirisena, Sunil Kumar Dubey, Ramesh Kumar, Jatin Shrinet, Sujatha Sunil

**Affiliations:** Vector Borne Diseases Group, International Centre for Genetic Engineering and Biotechnology (ICGEB), New Delhi-110067, India; ankitkumar.bcas@gmail.com (A.K.); psibms20@gmail.com (P.S.); nilupamali@gmail.com (P.S.); sunil1986dubey@gmail.com (S.K.D.); kumar.ramesh475@gmail.com (R.K.); jatbioinfo@gmail.com (J.S.)

**Keywords:** mosquitoes, innate immunity, pathogens, signaling pathways, RNA interference

## Abstract

Mosquitoes live under the endless threat of infections from different kinds of pathogens such as bacteria, parasites, and viruses. The mosquito defends itself by employing both physical and physiological barriers that resist the entry of the pathogen and the subsequent establishment of the pathogen within the mosquito. However, if the pathogen does gain entry into the insect, the insect mounts a vigorous innate cellular and humoral immune response against the pathogen, thereby limiting the pathogen’s propagation to nonpathogenic levels. This happens through three major mechanisms: phagocytosis, melanization, and lysis. During these processes, various signaling pathways that engage intense mosquito–pathogen interactions are activated. A critical overview of the mosquito immune system and latest information about the interaction between mosquitoes and pathogens are provided in this review. The conserved, innate immune pathways and specific anti-pathogenic strategies in mosquito midgut, hemolymph, salivary gland, and neural tissues for the control of pathogen propagation are discussed in detail.

## 1. Introduction

Mosquitoes are an immense public health concern owing to their role in transmitting diseases. However, they are constantly threatened by the invasion of microorganisms. Their tightly closed, hydrophobic outer cuticle serves to protect the internal organs of the mosquitoes from the outside environment as well as the entry of pathogens. When pathogens do enter the mosquito due to an accidental break in the outer cuticle, several features such as coagulation, melanization, hemocyte degranulation, and scar formation are activated to prevent pathogen entry [1,2]. Additionally, mosquitoes use their innate immune system, comprising of a cellular component and a humoral component, to fight pathogens once they enter [3]. Hemocytes are the main component of the cellular arm of immunity, whereas the soluble components in the hemolymph, such as pattern-recognition receptors (PRRs), antimicrobial peptides (AMPs), and components of the phenoloxidase cascade, form the humoral components of innate immunity [4,5,6].

Most pathogens enter the mosquitoes through the consumption of a blood meal, which is a physiological necessity for the female mosquitoes for their egg development [7]. Upon blood feeding, the pathogens pass into the lumen of the midgut. In the midgut, the pathogens face chemical and physical barriers that they need to overcome to establish themselves within the mosquito [8,9,10]. If the pathogens need to be orally transmitted, it is essential that they reach the salivary gland before or after proliferating inside the susceptible cells. If the infection route is transovarial, the pathogens need to gain access to the reproductive organs [11,12].

Once the mosquitoes consume a blood meal, a cascade of events is activated to circumvent the stress of the oversized unbalanced blood meal. As a first step, the toxic heme present in the blood that is ingested into peritrophins is trapped within a peritrophic matrix, making it less toxic; it becomes aggregated, and is excreted with the feces [13]. Alongside, numerous molecular events are also triggered: genes responsible for the secretion of hydrolytic enzymes, peritrophic matrix, extracellular matrix proteins such as peritrophins and mucins, iron-responsive genes, and lipid metabolism are regulated [14,15,16]. Upregulation of antioxidant response proteins takes place upon the consumption of a blood meal [14,17]. Additionally, a blood meal elicits an immune response in the mosquito; however, it has been observed that if a blood meal is infected with a pathogen, there is an enhanced surge of immune effector molecules [18,19,20,21,22,23]. Specifically, there is a tissue-specific regulation of these effector molecules, as seen in mosquito genera such as *Anopheles* and *Aedes* [24,25]. In essence, barrier-mediated temporary compartmentalization in the mosquitoes helps to block the pathogens in the initial stages and pathogen-recognition receptors and an array of molecular mechanisms help to minimize the establishment of pathogens. At the same time, it is essential for the mosquitoes to manage and minimize immune activation and maintain a relatively low immunity for the survival and maintenance of commensal microbes in order to maintain a balance.

This review describes the various phenomena that the mosquito utilizes for combating pathogens, such as the physical and physiological barriers that prevent pathogens from entering the body and the downstream proceedings like signaling, modulation, and effectors of immune pathways that play a vital role in mosquito innate immunity. A literature search using a combination of different keywords resulted in a total of 1990 articles ( Figure S1). After removing the duplicates, 1671 articles were obtained. Abstracts of these articles were screened, and 431 articles were further excluded from the study. A total of 796 full-text articles were distributed among the authors for further screening, and after discussions, 549 articles were excluded on the basis of repeated information, irrelevant data, and experiments related to organisms other than arthropods. Information from 247 articles was finally used for this review.

## 2. The Immune System of Mosquitoes

Mosquitoes possess physical barriers such as a hard exoskeleton, and they lack an adaptive immune system, unlike higher organisms. Mosquitoes entirely depend on their innate immune system to fight infections caused by pathogens such as viruses, bacteria, fungi, and parasites [26,27,28].

### 2.1. Physical Barriers

The first level of the insect immune system comprises of the cuticular and epithelial barriers—the epidermal, intestinal, and tracheal networks. From here, the systemic response may spread through the hemolymph (an open circulatory system that fills the hemocele). The humoral and cellular responses in the hemocele, midgut, and salivary gland allow the mosquitoes to rapidly respond to infections. All three compartments are briefly described in the following sections.

#### 2.1.1. Midgut

The midgut comprises of a narrow anterior region involved in sugar absorption and a wider posterior region involved in blood absorption. The anterior part has microvilli, a smooth endoplasmic reticulum, and a well-developed basal labyrinth, whereas the posterior part has a noticeable rough endoplasmic reticulum (RER) and a high number of mitochondria. Upon acquisition of a blood meal, drastic changes have been observed in the midgut epithelium, including condensation of nuclei, enlargement of mitochondria, and formation of concentric whorls in the RER [29,30,31,32]. The peritrophic matrix, which is a chitinous sac, is secreted by the epithelium into the midgut to facilitate blood meal digestion. It is important for the pathogens in a blood meal to enter the epithelial cells through the microvilli before they are digested. An earlier study conducted in mosquitoes has shown the presence of a distinct peritrophic matrix following an infectious blood meal [33].

#### 2.1.2. Hemocele

Pathogens need to cross the hemocele or the open body cavity of the mosquitoes to enter the salivary gland or other tissues; at this juncture, the pathogens are exposed to the mosquitoes’ immune system [34]. The hemocele contains all visceral organs and is outlined by the outer cuticle and basal lamina [1,2]. According to Jonas and colleagues, the hemolymph current affects the temporal and spatial controls of the antipathogen response. They also hypothesized that synchronized interactions between the insect’s open circulatory system and immune system are vital for an efficient insect immune response [35]. Pathogens enter the hemocele either by ingestion or by penetration and are then disseminated throughout the body by the natural flow of the hemolymph or pathogen-derived active motility [36,37,38]. In the hemocele, hemocytes, humoral immune factors, and pathogens exist close to each other along with the mosquito’s circulatory organs. According to recent studies, upon infection, hemocytes travel to the periostial areas, where they form a key component of the cellular immune response. Hemocytes secrete humoral immune factors involved in the killing of pathogens through the production of PRRs and proteins responsible for phagocytosis, nodulation, and other molecules such as melanization modulators and enzymes, signal transduction proteins, stress response proteins, and AMPs [39,40,41,42,43].

#### 2.1.3. Salivary Glands

The salivary glands of mosquitoes play a major role in disease transmission. In female mosquitoes, salivary glands consist of three lobes connected by a main salivary duct [44,45,46]. Entry into the salivary gland is important for a majority of pathogens to complete their life cycle [47,48,49]. Salivary glands secrete various proteins participating in different activities, such as lectins, which are involved in sugar feeding, apyrase which are involved in blood feeding, and D7 which interfere with hemostatic and vertebrate immune responses [50]. The salivary lobes are encircled by epithelial cells and are bound by the basal lamina. The epithelial layer forms a physical barrier for the pathogens [51]. Many studies have shown that arboviruses such as dengue virus-2 (DENV2) and chikungunya virus (CHIKV) infect the proximal, lateral, and median lobes, whereas the Sindbis virus (SINV) does not seem to infect the median lobe in *Aedes albopictus* or *Aedes aegypti*; [12,52,53,54,55,56,57]. It is hypothesized that Flavivirus replication occurring in the smooth membrane structures of the salivary gland and virus-induced convoluted membranes and tubular proliferated membranes, which are hypothesized to be alternate and reversible structures, help in pathogen entry [58,59,60,61] whereas *Plasmodium* and other pathogens have evolved to bind to some specific surface factors. Although experimental evidence to prove the active role of salivary gland as an immune organ is lacking, the saliva contains complex protein–peptide mixtures, antimicrobials, antihemostatics, proteins with angiogenic or antiinflammatory properties, and immune modulators, which are injected into the host along with the pathogens [62,63].

### 2.2. Physiological Barriers to Protect Mosquitoes against Pathogens

**Figure 1:**
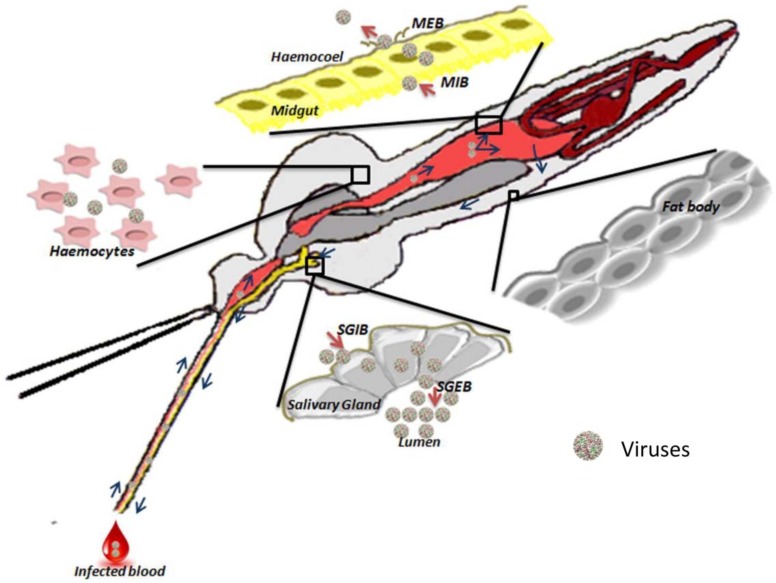
The schematic representation of immune responses using physical and physiological barriers upon infection in mosquitoes. MIB: midgut-infection barrier (pathogens establish an infection in the midgut epithelium and replicate in the midgut epithelial cells); MEB: midgut-escape barrier (pathogens pass through the basal lamina and replicate in other organs and tissues); SGIB: salivary gland infection barrier; SGEB: salivary gland escape barrier (these transmission barriers infect the salivary gland and escape into the lumen of the salivary gland).

Physiological barriers are processes that occur in the mosquito body in response to pathogens. Depending on the locale and the state of infection, different responses occur in the mosquito, which are aimed to eliminate the pathogens from the system. It is important for a pathogen to defeat both the physical and the physiological barriers in order to successfully establish infection (Figure 1).

#### 2.2.1. Midgut-Infection Barrier

In refractory mosquitoes with a midgut-infection barrier (MIB), pathogens cannot infect and/or replicate in the mosquito midgut cells. This may be due to the following reasons: a lack of cell surface receptors for the pathogens to initiate infection, the midgut cells possibly being nonpermissive to infection, or strong immune response against pathogen replication. Several hypotheses explain the above-mentioned mechanisms that lead to MIB: diversion of the blood and pathogens into the mosquito crop that has a chitin-lined sac; compartmentalization of pathogens, such as viruses, by the peritrophic matrix [64]; digestion of the pathogens by enzymes in the midgut, leading to their inactivation; pathogen–midgut interactions that prevent binding [10,65]; and the absence of cell surface receptors in midgut epithelia [10].

#### 2.2.2. Midgut-Escape Barrier

In the case of midgut-escape barrier (MEB), potential vectors may allow pathogens to replicate in the midgut, even to high titers, but the virus may not be able to exit the midgut and disseminate infection. Researchers have conducted electron microscopy and histochemistry studies to understand how pathogens escape the midgut and infect the other tissues of the mosquitoes [66]. Factors such as pathogen load or the dose and timing of the transit of the pathogen seem to determine the successful escape of the pathogen from the midgut [28]. Two main hypotheses explain the MEB: direct passage through the basal lamina and use of tracheal cells as a channel between the midgut and the hemocele [28]. MEB was occasionally found to be dose-dependent. Studies on western equine encephalitis virus (WEEV) show the dose dependency, and these arboviruses seem to have genetically controlled mechanisms that can modulate the midgut of the mosquito [67]. Similarly, Rift Valley fever virus (RVFV) in *Culex pipiens* and Venezuelan equine encephalitis virus (VEEV) in *Culex taeniopus* have shown dose-independent MEB. It is possible that dose-independent MEB comprises structures that separate the midgut from the hemocele and basal lamina, which the pathogens are incapable of crossing. A study based on *An. gambiae* reported that an epithelial serine protease (AgESP) plays a central role in the traversal of *Plasmodium* parasites across the midgut epithelial barrier [68]. Another study conducted on *An. gambiae* observed that ookinetes disrupt the midgut epithelial barrier to escape the midgut, during which injured epithelial cells came in contact with gut microbiota. This stimulated the mosquito immune system and triggered the differentiation of hemocytes into the granulocytes in hemocoel, which ultimately reduced the *Plasmodium* survival upon secondary infections. As upon re-invasion by the parasite a stronger antibacterial response was generated which affects the pathogens indirectly. It shows that the MEB also plays an important role in mosquito’s innate immune memory against pathogens [69].

#### 2.2.3. Salivary Gland Infection Barrier and Salivary Gland Escape Barrier

The molecular basis of the salivary gland infection barrier (SGIB) and salivary gland escape barrier (SGEB) is not well defined. It is believed that there are dose-dependent and dose-independent transmissions of pathogens in different virus–mosquito combinations, including La Crosse Virus (LACV) in *Aedes triseriatus*, eastern equine encephalitis virus (EEEV) in *Ae. albopictus*, and Japanese encephalitis virus and West Nile virus in *Cx. pipiens* [70,71,72]. SGIB has been demonstrated in WEEV in *Culex tarsalis* mosquitoes [67] and in EEEV. These viruses disseminated from the midgut to the fat body but could not enter the salivary glands. Experiments conducted using RVFV in *Aedes* spp. provide evidence of the basal lamina surrounding the salivary gland acting as a major infection barrier [73]. Moreover, using baculovirus in *Bombyx mori*, researchers have found that the basal lamina prevents the penetration of budding virions into the salivary gland [74]. The role of hemoplymy in SGIB was described by Hardy and colleagues, according to whom the hemolymph of *Cx. tarsalis* was more vulnerable to WEEV infection when compared with females of the refractory strain [65]. SGEB has been reported in *Aedes* and *Culex* mosquitoes transmitting LACV, SINV, and RVFV [75,76,77,78]. In the case of parasites, which need to modify the actin cytoskeleton of the epithelial barrier of salivary glands, it was found that AgESP aids the escape of *Plasmodium* through the salivary gland epithelial barrier in *An. gambiae* [68].

### 2.3. Molecular Basis of Immunity

#### 2.3.1. Recognition

Whenever a pathogen invades a mosquito, it encounters several host-derived molecules that interact with these foreign agents depending on their structure and surface molecules. These host-derived molecules are called PRRs, which bind to pathogen-associated molecular patterns (PAMPs). Generally, PRRs are secreted proteins that are found in different parts of the body (midgut and hemocele). In an in silico study conducted using the genome of *Anopheles gambiae*, approximately 150 putative PRRs were identified, the majority of which were found to be secreted proteins having adhesive domains to interact with PAMPs. These PRRs were found to cluster as members of large families of genes [5]. Experimental results suggested that many of these PRRs are involved in immune responses against different foreign microbes, although their exact role is not clear as their corresponding PAMPs have not been identified [79]. Several families of proteins function as PRRs, one of these being the thioester-containing proteins (TEPs). These proteins are generally found in the hemolymph, and their role has been experimentally reported in *D. melanogaster*, *An. gambiae*, and *Ae. aegypti* as an essential pathogen-recognition molecule leading to the neutralization of the pathogen [4,80,81]. One of the well-studied proteins of this family is TEP1, which is produced by hemocytes and functions as a phagocytosis enhancer. It is secreted in the hemolymph as a single chain peptide that is inactive and is activated by proteolytic cleavage [6]. The activated TEP1 protein is then stabilized by the formation of a leucine-rich repeat complex containing LRIM1 and APL1C proteins; only after the formation of this complex does TEP1 bind to the bacteria in the hemolymph and the *Plasmodium* (ookinetes) in the midgut, leading to their destruction [82,83]. The role of LRIM1 and APLC1 in the anti-*Plasmodium* response in *Anopheles* has been studied in detail. Silencing of these genes has been reported to lead to the altered immune response against *Plasmodium* infection [83]. A genome-wide study showed that susceptibility and resistance to *Plasmodium* infection varied depending upon the variations or polymorphisms found in the sequences of LRIM1 and APL1C proteins [84]. In another study conducted on *Ae. aegypti* mosquitoes, the role of TEPs was assessed upon flaviviral (DENV) and West Nile virus (WNV) infections. In this experiment, TEP1 and TEP3 were bioinformatically selected and RNA interference (RNAi)-mediated modifications were made in vivo, including over-expression and truncation of these two proteins, respectively. It was found that when TEP1 was over-expressed, the viral load was reduced; however, over-expression of TEP3 did not lead to a reduction of viral load, thereby confirming the role of TEP1 in regulating viral infection [80]. Another essential family of proteins which plays a central role in the mosquito’s innate immune response by recognition of PAMPs is the fibrinogen-related protein family (FREP). In *An. gambiae,* it has been reported as the largest pattern-recognition protein family consisting of 59 putative members, most of which showed immune responsive transcription when challenged by infection with bacteria, fungi, or *Plasmodium* [33,85]. Using RNAi-mediated gene-silencing assays, it was observed that members of the FREP family play a central role in the innate immune response and maintenance of immune homeostasis in mosquitoes [86]. One of the most studied and effective members of this family is fibrinogen immunolectin 9 (FBN9), which interacts with both Gram-positive and Gram-negative bacteria; its colocalization with *Plasmodium* has also been reported in midgut epithelial cells, suggesting that it binds to *Plasmodium* directly, leading to their destruction [19,87,88]. C-type lectins are another family of proteins playing an important role in the recognition of pathogens. These are membrane-bound or soluble proteins which bind in a calcium-dependent manner to carbohydrates. C-type lectins have been found to have both positive and negative impacts on immune response against pathogens. In *An. gambiae*, two members of this family, CTLMA2 and CTL4, serve as inhibitors of melanization in the midgut, whereas in the hemocele, the same C-type lectins are present as disulfide-linked heterodimers killing *Escherichia coli* in a melanization-independent manner. RNAi-mediated silencing or knockdown of either of these proteins leads to increased bacterial loads in the hemocele, causing mortality, which indicates that these two proteins are essential for the antibacterial response in the mosquito [89,90].

Gram-negative binding proteins (GNBPs) are another family which plays an important role in the recognition of bacteria and parasites. Members of this family are found in different tissues, such as hemocytes, midgut, and salivary glands and are found to be over-expressed following the infection of bacteria and/or *Plasmodium* [91]. In *An. gambiae*, six members of this family are considered to be PRRs which bind to β-1,3-glucan and lipopolysaccharide found on the surface of pathogens. All members of this family are found to be upregulated upon infection, but they vary in their antimicrobial specific activities. For example, GNBP4 plays an important role in neutralizing *E. coli*, *Staphylococcus aureus*, and *Plasmodium berghei* but not *Plasmodium falciparum*. Whereas GNBPA2 is an essential component involved in killing *E. coli* and *P. falciparum*, it shows insignificant activity against *P. berghei* and is not effective against *S. aureus* [92].

#### 2.3.2. Signaling

**Figure 2:**
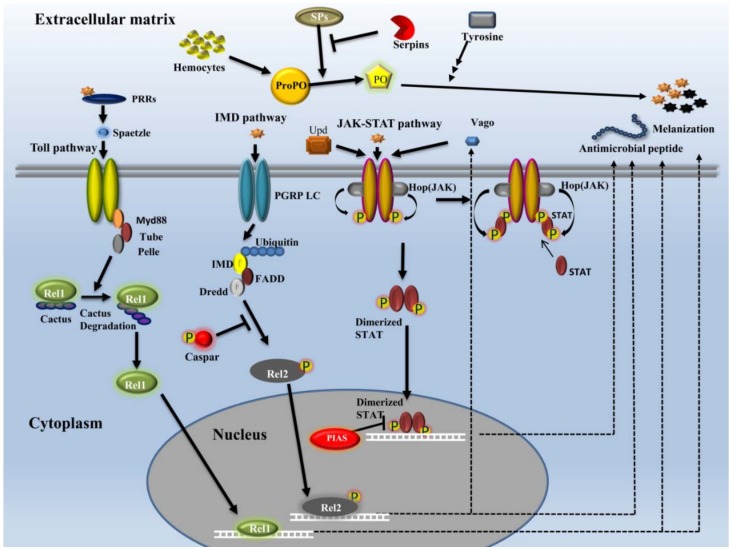
The signaling pathway of mosquito’s innate immunity. Recognition of pathogen-associated molecular patterns by pattern-recognition receptors (PRRs) activates the Toll pathway by proteolytic cleavage of spaetzle which binds to Toll receptors and triggers the signalling through adaptor proteins. This results in phosphorylation and degradation of the Cactus protein, which is an inhibitor of Rel1. Degradation of Cactus allows Rel1 to be translocated to the nucleus for the activation of transcription of genes regulated by Toll pathway. The IMD pathway is triggered by the binding of pathogens with PGRP-LC, which further triggers the signalling through IMD, FADD, and Dredd. This results in the phosphorylation of Rel2 by the activity of the Dredd protein. This step is regulated by the Caspar protein which is a negative regulator of the IMD pathway. Now Rel2 enters the nucleus to regulate the transcription of IMD-regulated genes, which are also involved in the synthesis of antimicrobial peptides. The JAK-STAT pathway is triggered by the binding of Upd with receptor proteins (Dome), leading to the activation of Hop proteins. Activated Hop proteins phosphorylate each other and STAT proteins, which are dimerized upon phosphorylation. This dimer of STAT proteins translocates to the nucleus and activates the transcription of JAK-STAT regulated genes, some of which are involved in the synthesis of antimicrobial peptides. PIAS is a negative regulator of dimerized STAT proteins inside the nucleus, and over-expression of PIAS inhibits JAK-STAT signalling pathway. Melanization is triggered by the activity of hemocytes, which is induced by the recognition of pathogens by PRRs. This results in the cleavage of prophenoloxidases (ProPO) by the activity of serine proteases (SPs), to form phenoloxidases (PO). Here, the activity of serine proteases is negatively regulated by serpins. Activated phenoloxidases trigger a series of reactions initiated in the presence of Tyrosine, ultimately leading to the melanization of invading pathogens.

The immune-signaling pathways protect mosquitoes from continuous exposure to the invading pathogens and opportunistic microbes as well as regulate the natural microbiota, for example, the gut flora. Many studies have reported the role of natural microbiota in enhancing the immune response and providing resistance against invading pathogens in mosquitoes [33,88,93,94]. Upon recognition of foreign pathogens by the PAMP receptors, the pathogens could be destroyed by the action of constitutive effector mechanisms such as melanization. Additionally, the induction of the immune-signaling pathways may lead to the production of AMPs, which neutralizes the invading pathogens. Three of these major pathways are the Toll pathway, the IMD pathway, and the JAK-STAT pathway. These pathways are activated and trigger effector molecules to neutralize the invading pathogens. They have been shown to be activated by several pathogens such as Gram-positive bacteria, fungi, *Plasmodium*, and viruses (Figure 2).

- *Toll pathway*

The Toll pathway was first identified during the genetic screening of genes involved in the early embryonic development of *Drosophila melanogaster*. The Toll pathway cascade of *Drosophila* reshaped the understanding of immune system not just in *Drosophila* but also in other insects, even the mammalian systems. In flies, the Toll pathway is essential in embryonic development and immunity [95]. The Toll pathway is induced by Gram-positive bacteria or fungi and activates cellular immunity and production of AMPs [96,97,98]. Toll receptors are activated upon the binding of proteolytically cleaved Spaetzle ligand, which eventually leads to the activation of NF-kB factors [99,100]. The molecular mechanism of the Toll pathway has been well studied in mosquitoes. Genes of the Toll pathway are controlled by Rel1, which is an NF-kB transcription factor and a homologue of Dorsal that plays a central role in the regulation of Toll pathway in *Drosophila*. Rel1 has two isoforms, namely, Rel1-A and Rel1-B, which are induced by septic injuries in larval or female mosquitoes [101]. Rel1 plays an essential role in the regulation of antifungal immune signaling through the Toll pathway. In a study based on *Ae. aegypti*, the RNA interference technique was used to generate two different transgenic mosquito lines. In one of them, Rel1 was over-expressed, whereas in the other Rel1 was knocked down. Here, it was noticed that over-expression of Rel1-A homology domain led to the over-expression of Rel1-B as well, which showed that Rel1-A Rel homology domain is actually regulating the transcriptional activation of Rel1-A and Rel1-B. This regulatory mechanism needs to be studied in greater detail. Additionally, it was seen that the over-expression of Rel1-A and Rel1-B leads to the activation of Spaetzle 1A and Serpin-27A, which otherwise occurs in the case of any fungal infection and/or septic injury. Importantly, the Rel1 over-expressed strain showed decreased susceptibility to pathogenic fungal (*Beauveria bassiana*) infections. On the other hand, the Rel1 knockdown strain showed increased susceptibility to fungal infections and diminished induction of Spaetzle 1A and Serpin-27A [102]. Further, studies have shown that proteolytic cleavage of Spaetzle is essential for the establishment of Toll signaling [103,104]. Serpin-27A regulates the melanization cascade by the inhibition of the prophenoloxidases activating enzyme in *Drosophila* and was found to be over-expressed by heat-killed bacteria in Aag-2 cells and by fungal infection in female *Ae. aegypti* mosquitoes [101,105]. Another molecule that functions as a negative regulator of Rel1, Cactus, helps the pathogen to survive by suppressing the activity of Rel1 [102]. It is known that the Toll pathway has a significant role in regulating resistance against the dengue virus in *Ae. aegypti*, which was concluded on the basis of the observation of post-infection regulation and functional assessment of various genes involved in the Toll pathway [94]. RNAi-mediated silencing of Cactus was reported to enhance the expression of AMP gene DEF (defensin), which aided in the control/neutralization of the dengue virus in *Ae. aegypti* [94]. In another experiment, when Cactus was silenced, the intensity of infection reduced in the *Anopheles* mosquitoes. On the other hand, when both Rel1 and Cactus were silenced in the mosquitoes, they were found to be more susceptible to infection [106].

- *IMD pathway*

The IMD pathway is another major signaling pathway that plays an essential role in mosquito immunity. Like the Toll pathway, the IMD pathway was also identified and studied in *Drosophila* for the first time, and since then it has been found to play an essential role in the immune system of insects as well as mammals. This pathway has molecules overlapping with that of the Toll pathway in eliciting an immune response.

As in other immune-signaling pathways, induction of the IMD pathway begins with microbial recognition by specific genome-encoded host-derived pattern-recognition molecules that bind to specific conserved structures present in the pathogens but not in the hosts. The activation of the IMD pathway has been reported by bacteria and *Plasmodium*, and an indirect effect of the IMD pathway has been shown on viral load in *Aedes* mosquitoes [107]. Although the Toll and JAK-STAT pathways control immune response against *Plasmodium*, the IMD pathway has emerged as the most effective pathway involved in immune response against the human malaria parasite [108]. Like Rel1 in the Toll pathway, Rel2, another molecule belonging to the same family, plays the central role in IMD signaling and regulates a major AMP, cecropin1. The *Rel2* gene produces two isoform proteins by alternative splicing: a full-length (Rel2-F) protein and a shorter one (*Rel2*-S). The shorter *Rel2*-S lacks the inhibitory ankyrin repeats and death domain [109]. Gene knockdown experiments showed that, in comparison to Relish found in *Drosophila*, which responds only to Gram-negative bacteria, both isoforms of Rel2 in *Anopheles* are involved in immune defense against the Gram-positive and the Gram-negative bacteria, respectively [109]. Rel2-F also modulates the intensity of infection in the vector with the malarial parasite *P. berghei* [109]. There is another protein, Caspar, which functions as a negative regulator of Rel2, similar to the function of Cactus for Rel1. Multiple studies have shown that Caspar functions as a negative regulator of the IMD pathway and that the immune response against *Plasmodium* could be exaggerated by the silencing of the *Caspar* gene [108]. Over-expression of the gene encoding Rel2 transcription factor confers complete resistance against laboratory-cultured *P. falciparum* in *An. gambiae*, *Anopheles stephensi*, and *Anopheles albimanus* mosquitoes [110]. A study conducted on *An. gambiae* infected with isolates of *P. falciparum* revealed the requirement of the PGRP-LC receptor which activates the IMD pathway, thereby emphasizing the role of the IMD pathway in *Anopheles* immunity against *Plasmodium* [110,111]. A global gene regulation study using Caspar-silenced mosquitoes having over-activated IMD pathway suggested that TEP1, FBN9, and a leucine-rich repeat family member (LRRD7/APL2) are key players in the defense against *Plasmodium* [112]. Another member of the leucine-rich repeat family, APL1A, was identified as an anti-*Plasmodium* effector controlled by Rel2 in the Ngousso strain of *An. gambiae* [113]. One of the distinct features of the IMD pathway is its activation, which is regulated by the endogenous bacterial flora of the mosquito midgut [107]. These bacteria exhibit a physiological role in the development, digestion, nutrition, and reproduction of the mosquito [114]. According to recent studies, mosquito microbiota have been found to have a profound effect on the immune system [93,113]. In another study conducted on *Ae. aegypti* mosquitoes infected with the DENV, a reciprocal tripartite interaction between the microbiota, immune system, and dengue virus infection was reported after the blood intake [93]. It is hypothesized that this kind of interaction between the three players may not be restricted only to DENV but could be a general feature of other arboviral interactions as well.

- *JAK-STAT pathway*

The Janus kinase/signal transducers and activators of transcription (JAK-STAT) pathway is known to be a major signaling pathway induced by interferons and are known to transcriptionally regulate genes involved in immune systems of vertebrates. In insects, it was first identified in *Drosophila* while studying its developmental aspects, and subsequently it was identified as an integral part of the antiviral response mechanism in *Drosophila* [115]. The major components of the JAK-STAT pathway are Unpaired (Upd) peptide ligand, transmembrane protein receptor (Dome), Janus kinase (JAK), and STAT proteins. The JAK-STAT pathway is induced by the binding of Upd to the extracellular terminal of the Dome receptors, followed by conformational modifications, such as the dimerization of these receptors, leading to the phosphorylation of Janus kinases associated with receptor dimers. The activated Janus kinase then phosphorylates the C-terminal side of the receptor dimers, producing binding pockets for STAT proteins. STAT proteins then bind to these pockets and are phosphorylated by the JAK–Dome complex, which results in the activation and dimerization of the STAT proteins, which are further translocated to the nucleus for transcriptionally regulating the expression of target genes [116]. The role of the JAK-STAT pathway in mosquito immunity was first discovered in *An. gambiae* by infecting them with bacteria (*E. coli* and *Micrococcus luteus*), which showed the translocation of the STAT protein to the nucleus [117]. In the case of *Ae. aegypti* mosquitoes, DENV replication in the midgut of mosquitoes was studied upon modulation of the JAK-STAT pathway using gene-silencing approaches. Silencing the inhibitor protein for activated STAT showed decreased replication of DENV in mosquito midgut, whereas knockdown of Dome receptors or Hop protein (homologue of JAK) resulted in enhanced viral replication in the midgut [118]. These results suggest that the JAK-STAT pathway plays an essential role in antiviral defense in mosquitoes as well, which also implies that this pathway is conserved in both vertebrates and invertebrates, including in insects [81,117,118,119].

- *RNAi*

Antiviral response as an innate immunity in insects is majorly regulated by the RNAi pathway. RNAi is a conserved sequence-specific gene-silencing mechanism that controls a myriad of functions in maintaining cellular homeostasis during pathogen infections. RNAi pathways include the generation of small RNA molecules of different characteristics, such as small endogenous interfering RNAs (siRNAs), microRNAs (miRNAs), and P element-induced wimpy testis (PIWI)-interacting RNAs (piRNAs), and interaction of these molecules with the RNA-silencing complex (RISC) to elucidate a defense response. Apart from the above-mentioned molecules, virus-derived exogenous small-interfering RNAs (siRNAs) may also initiate the antiviral response. Many viruses produce viral dsRNAs that serve as replication intermediates inducing antiviral response in insects. Among all the pathways of RNAi, the siRNA pathway is the main pathway for the antiviral response. Owing to its importance in insect immunity, this phenomenon will be discussed in detail in a later section of this review.

#### 2.3.3. Modulation

In mosquitoes, the immune response is modulated by a family of proteins that regulate and manipulate the signaling pathways that are triggered upon recognition of pathogens. Modulators facilitate the amplification of recognition signals to induce different effector mechanisms to neutralize the invading pathogens. Serine proteases (SP) are the major modulators of the immune system that are activated by a series of proteolytic cascade events upon recognition of pathogens [120]. These molecules play an important role in the coagulation of proteins in the hemolymph, synthesis of antimicrobial peptides (AMPs), melanization of pathogens, and digestion of food [121,122]. The first serine protease (ISP13) was discovered in *An. gambiae*; upon evaluation of its involvement in the immune system of a mosquito, it was found to be expressed mainly in the midgut in response to bacterial and *Plasmodium* infections [91,123]. Additionally, studies have revealed the presence of two more serine proteases having N-terminal CLIP domains. These two SPs, CLIPB14 and CLIPB15, are synthesized by the hemocytes and are released into the hemolymph. These SPs appear to play an important role in enhancing the extent of melanization upon bacterial and *Plasmodium* infections [124,125]. Another class of molecules that regulate the activity of serine proteases and manipulate the immune system of mosquitoes are the serine protease inhibitors (serpins) [126]. Different serpins play a variety of roles in the mosquito immune system. Gene knockdown studies of a serpin (SRPN6) in *An. stephensi* and *An. gambiae* showed an increased number of parasites in *An. stephensi* and enhanced melanization of parasites in *An. gambiae*. This study also suggested that SRPN6 is a component of the midgut epithelial immune system, which acts synergistically with C-type lectin protein (CTL4) [127].

#### 2.3.4. Effectors

- *AMPs*

AMPs are small peptides that are mostly positively charged and are produced in hemocytes, fat bodies, and epithelial cells in response to signals received through signaling pathways upon recognition of any pathogen or foreign agent. These are mainly produced in response to PRRs and other recognition machinery present in the insect. After being synthesized in the above-mentioned cells, AMPs are transported to the hemolymph, where they reach higher concentrations, and are then transported to the sites of action. There are several classes of AMPs, categorized on the basis of their structure, function, and specificity. In *Ae. aegypti*, five different classes have been identified, namely, defensins, cecropins, gambicin, diptericin, and attacins [128,129,130,131]. These AMPs are present in other mosquito species as well, such as *An. gambiae* [132]. All these five classes of AMPs have shown activities against different specific pathogens. Defensin is the predominant, immune-inducible peptide in mosquitoes, which shows antibacterial activity against Gram-positive and Gram-negative bacteria; its expression was also seen upon infection with filarial worms in *Ae. aegypti* [130,132,133]. Defensins were identified in a very interesting manner. When *Ae. aegypti* mosquitoes were injected with *E. coli* and *M. luteus*, an antibacterial response was generated in the hemolymph. Three novel peptides that were not expressed in naive mosquitoes were isolated and checked for antibacterial activity, and they were found to be antibacterial against Gram-positive bacteria, whereas one of them also showed antibacterial activity against Gram-negative bacteria [134]. In the case of *An. gambiae*, however, it has been reported that defensin does not show anti-*Plasmodium* activity [135]. However, cercopin A (cecA) has been shown to restrict Plasmodial infection in *An. gambiae.* In a study on *An. gambiae,* recombinant DNA technology was utilized to make a transgenic line over-expressing cecA upon blood feeding. It was observed that there was a 60% reduction in the number of oocysts in transgenic mosquitoes than in the nontransgenic ones, upon infection with *Plasmodium berghei*. This is a study that paved the way to devise a method based on recombinant DNA technology for controlling vector-borne pathogens by reducing the capacity of the mosquitoes to serve as a vector [131]. A recent study found that expressions of cecA, D, E, F, and N are also stimulated by *E. coli* and *S. marcescens* in Aag2 cells [136]. Gambicin has also shown anti-microbial/-plasmodial activity. Silencing of the *gambicin* gene resulted in an increased *P. berghei* load in *An. gambiae* [137]. Additionally, a study conducted on Aag2 cells showed that silencing of the key components of JAK-STAT pathway leads to a reduced activity of gambicin against bacterial infections, showing the central role of immune signaling in AMP production [136]. In another study, overall seventeen AMPs belonging to the above-mentioned classes were identified by carrying out sequence analysis of *Ae. aegypti.* It was found that mRNA expression of seven AMPs was significantly enhanced upon DENV-2 infection [131]. Expression of diptericin and attacin was not found to be significantly changed upon microbial infections in Aag2 cells [136]. However, significant enhancement of attacin expression was observed in *Ae. aegypti* upon DENV-2 infection and a slight increase in the expression levels of diptericin was also seen upon DENV-2 infection in the same study [131].

- *Reactive oxygen species/reactive nitrogen species*

Reactive nitrogen species (RNS) and reactive oxygen species (ROS) are two very important effector components of the mosquito immune response. Their role has been well described in the case of bacterial and plasmodial infections. When these pathogens are ingested by the mosquitoes through an infected blood meal, a series of reactions are induced, leading to the activation of this system. Multiple studies have reported that ROS levels increased upon infection with *Plasmodium* in a particular strain of *An. gambiae* that was refractory to *Plasmodium* infection [138]. Another study reported that oral administration of antioxidants reduced melanization of *Plasmodium* [139]. These studies reveal that ROS is essential for mosquitoes to mount an effective immune response against *Plasmodium* and bacteria. It appears that ROS attacks *Plasmodium* ookinetes in the midgut and bacteria inside hemocele. In the case of *Plasmodium*, it has been well documented that ROS kills through melanization and lysis [138]. In a study conducted with *An. gambiae* mosquitoes, it was found that L3-5, a strain resistant to *P. berghei*, lives under constant oxidative stress, which promotes the melanization of *Plasmodium* ookinetes as they pass through the midgut epithelium. The susceptible strain G3, however, kills ookinetes by a lytic mechanism based on oxidative stress induced upon infection, which is maintained by the suppression of catalase, the enzyme which converts hydrogen peroxide into oxygen and water [139]. RNS includes nitric oxide, a free radical produced during the oxidation of l-arginine by an enzyme called nitric oxide synthase to l-citrulline. In *Anopheles* mosquitoes, nitric oxide synthase is a single-copy gene having 18–22 transcripts, out of which three are induced by *Plasmodium* infections and another by bacterial infections [140,141,142]. Inside the midgut of mosquitoes, *Plasmodium* glycosylphosphatidylinositols and *Plasmodium*-derived hemozoin ingested with the blood meal induce the transcription of nitric oxide synthase through the STAT pathway, and the nitric oxide produced then neutralizes ookinetes by lysis [25,117,143,144]. Expression of nitric oxide synthase and heme peroxidase 2 (Hpx2) is induced by midgut epithelial cells upon *Plasmodium* infection. Hpx2 is a mediator of nitration that potentiates the toxicity of NOS by promoting nitration in the midgut [145,146]. Another component of this nitration response is NADPH oxidase 5 that serves as a source of hydrogen peroxide, which is essential for Hpx2 to remain active [145]. In the case of bacteria, upon infection with *E. coli*, nitric oxide synthase is transcriptionally upregulated inside the hemocele for killing the bacteria [140].

- *Melanization*

Melanization in insects is another major effector of immune response against the invading pathogens. It is also involved in the formation of the hard protective layer around the egg chorion and in wound healing. As it is involved in both development and immune defense, melanization is a complex mechanism to study. Studies suggest that melanization involves the formation of a thick and dark proteinaceous complex around invading pathogens such as malarial parasites, fungi, and some bacteria. This proteinaceous complex is primarily composed of thick layers of melanin, which are formed inside the mosquito body upon recognition of specific pathogens by PRRs. Recognition of pathogens by PRRs trigger a series of reactions involving many enzymatic and non-enzymatic reactions [147].

In general, melanization, as studied in systems other than mosquitoes, begins with the hydroxylation of phenylalanine, which is catalyzed by phenylalanine hydroxylase in the presence of 6(*R*)-l-erythro-5,6,7,8-tetrahydrobiopterin and oxygen, to form tyrosine, which is the limiting factor in melanin production. Tyrosine is then hydroxylated by phenoloxidase (PO) to Dopa, and Dopa is further oxidized to dopaquinone. Dopaquinone either forms cysteinyl and glutathione conjugates in the presence of thiols, forming yellow–red pheomelanins, or in the absence of thiols, dopaquinone is spontaneously converted to dopachrome, which is further converted to dark brown-black polymer eumelanin [148].

The major components of melanization are POs, serine proteases, and serpins. There are at least 20 reported POs in different mosquito species, most of which are produced in the hemocytes, as reported in *Ae. aegypti* and *Armigeres subalbatus* [149,150,151]. All mosquito POs have six conserved histidine residues in two conserved copper-binding sites and a potential cleavage site for activation by proteolysis in between a phenylalanine–arginine linkage. Different POs might have different specific functions based on their sequence, structure, and localization. For example, in a gene-profiling study of POs in *An. gambiae* upon blood feeding, it was observed that after blood feeding, four PO genes were over-expressed whereas one was downregulated [152]. Similarly, in another study conducted on *Ar. subalbatus*, it was seen that one of the PO was specifically involved in the thickening of egg chorion after a blood meal [153]. Serine proteases are primarily involved in the activation of prophenoloxidases (proPOs) by proteolytic cleavage to form POs. Whereas SPs and POs are involved in melanization, serpins act as inhibitors of serine protease activity. In effect, serpins can inhibit melanization and are reported to control melanization partially or completely [154]. Melanization kills pathogens by restricting nutrition uptake from the surroundings due to the formation of a thick surrounding coat/layer. High oxidative stress is created during the production of melanin and its intermediates. This highly oxidative environment is lethal for pathogens; it is also harmful to the mosquito cells, leading to damage and causing negative effects. In many organisms, oxidative stress is neutralized by the activation of molecules such as glutathione reductase, an enzyme that catalyzes the reduction of glutathione disulfide to glutathione in an NADPH-dependent manner. However, this system has not been reported in mosquitoes. In *Anopheles* and *Ae. aegypti*, an NADPH-dependent thioredoxin reductase has been reported to serve the same purpose [155,156]. It remains to be proven that this molecule is involved in melanization. Melanization is an energy-expensive process, including a series of biochemical reactions occurring in response to a blood meal, wound/injury, or pathogen invasion along with its role in egg development, and poses a fitness cost to mosquitoes. Although several studies have established that melanization is a highly specific phenomenon that occurs only against specific bacteria, filarial worms, and *Plasmodium* species, a study conducted on *An. gambiae*, reported the induction of melanization through the inoculation of sephadex beads in mosquitoes and argued that this could be due to the surface characteristics of the beads. The study further showed that when a mosquito was overloaded with beads, it showed irregular melanization patterns, rendering some beads to be more strongly melanized than others. The authors suggested that the pathogen load might also regulate melanization to some extent and suggested that higher loads of pathogens might weaken the melanization response [157].

- *Apoptosis*

Apoptosis is a highly regulated process leading to programmed cell death in many organisms. It is essential for removing damaged and infected cells in order to maintain homeostasis and has been proposed as an antiviral defense mechanism in insects. Many viruses are known to have genes with antiapoptotic activity, inhibiting the apoptosis triggered during viral infection. Genetic studies on four apoptotic inhibitor antagonist genes, namely, *reaper*, hid, grim, and sickle, have provided insights into the molecular mechanism of apoptosis and into developmental cell death in an insect system. All these genes are regulated at a transcriptional level and in a programmed manner based on the requirement of developmental process in cells.

In mosquitoes, apoptosis has been reported to participate in the regulation of viral/pathogen loads. In *An. gambiae*, one apoptotic inhibitor antagonist gene, *michelob-x* (*mx*), was identified using an advanced bioinformatics approach. Although *mx* differed structurally from the apoptotic inhibitor antagonist genes found in *Drosophila*, its transcriptional regulation was found to have similarities with the *reaper* gene of *Drosophila* system [158]. Identification of the *mx* gene showed the possibility to verify the probable involvement of a *reaper*-like apoptotic inhibitor antagonist in regulating pro-apoptotic response against viruses. In a study conducted in an *Ae. aegypti* system, it was found that exposure of mosquito larvae to baculovirus CuniNPV (*Culex nigripalpus* nucleopolyhedrovirus) showed rapid induction of *mx*, which was seen in the midgut cells of viral-infected larvae. These infected cells went into a quick apoptotic cell death within 4–6 h post-infection. An interesting observation was that this rapid induction of apoptosis was seen only in *Ae aegypti* larvae, which are refractory for CuniNPV infection, whereas no such rapid induction of apoptosis was reported in the larvae of *Culex quinquefasciatus*, a species susceptible to CuniNPV infection. On the basis of this observation, it could be hypothesized that apoptosis plays an important role in mediating resistance against viral infections in mosquitoes [159].

There are two more important components of the apoptotic pathway in mosquitoes, namely, Aeiap1 and Aedronc. Whereas Aedronc is an initiator caspase, Aeiap1 functions as an inhibitor of apoptosis [159,160,161]. In a study conducted on laboratory-maintained *Ae. aegypti* mosquitoes, an evaluation of the role of apoptosis upon infection with SINV revealed that inducing apoptosis increased virus infectivity in the midgut and viral dissemination to the other parts of the body, whereas inhibition of apoptosis caused decreased viral infectivity and dissemination [162]. These results contrast with the hypothesis that apoptosis is an antiviral immune defense system in mosquitoes. It is possible that apoptosis damages the cells of physical barriers, which restricts the infectivity of invading viruses; when the apoptotic pathway is artificially induced, viral infectivity and dissemination are enhanced. So, different studies conducted on different combinations of viruses and vectors showed varied responses in apoptotic pathways, based on their genetic backgrounds.

- *Autophagy*

Autophagy is a well-known cellular process found in most of the vertebrates and invertebrates, in which degradation of damaged or unwanted cell organelles is induced by the formation of a double membrane structure due to the scarcity of nutrients or pathogenic infection. Most information on understanding the role of autophagy was accomplished using viruses. In a study conducted on *Ae. aegypti* mosquitoes, it was seen that upon DENV2 infection, Autophagy-related 5 (ATG5) transcript levels were elevated in the midguts of DENV2-susceptible mosquito strains in comparison to refractory strains, and this increase was found to coincide with the increase in the expression of genes involved in apoptosis [163]. Enhanced DENV2 viral load was observed in the mosquitoes when these genes were silenced, accompanied by the accumulation of Atg8-PE, where Atg8 is an induction and progression marker of autophagy and PE (phosphatidylethanolamine) aids in embedding Atg8 into the membranes of elongating phagophores [163]. These findings reveal that the apoptosis machinery regulates autophagy in a directly proportional manner.

- *Phagocytosis*

In mosquitoes, phagocytosis is an effector cellular process that effectively neutralizes *and* removes microorganisms such as bacteria, yeast, *Plasmodium*, and other minute abiotic particles from the host system [41,164,165]. It is initiated by the recognition of a microorganism or particle, which is then engulfed by a phagocytic cell forming a phagosome that carries that particle or microorganism. The phagosome, when internalized, fuses with the lysosome in the cytosol, and the microorganisms are neutralized by the hydrolytic enzymes present inside the lysosome. Reports have revealed that in mosquitoes, a subpopulation of hemocytes is phagocytic in nature and internalizes and kills the invading pathogens [42]. Phagocytosis is an important and complex antimicrobial defense mechanism in mosquitoes, and major regulators of phagocytosis were found to be PRRs, transmembrane receptors, and other signaling proteins. Among these, TEP1 and LRIM1 are the major recognition peptides that are involved in the phagocytic degradation of microorganisms, as they initiate this process by opsonizing the microorganisms [82]. AgDSCAM (hypervariable immunoglobin domain containing receptor of *Anopheles gambiae*) is another molecule that recognizes bacteria and initiates their phagocytosis by hemocytes. AgDSCAM is an essential part of antibacterial immunity as shown in a study that involved knockdown of AgDSCAM in mosquitoes, leading to enhanced bacterial growth in the hemocele [88]. Transmembrane receptors function by directly recognizing the microorganisms or by recognizing the ones opsonized by other hemocele proteins. Such receptors are β integrin (BINT2), a peptidoglycan recognition protein (PGRPLC), and lipoprotein receptor-related protein (LRP1) [166,167]. Other major components of the phagocytosis mechanism in mosquitoes are intracellular CED proteins, which trigger phagocytosis upon recognition of bacteria. In a study conducted on *An. gambiae*, knockdown of CED2, CED5, and CED6 reduced the phagocytic efficiency to a great extent, which showed their involvement and importance in antibacterial response in mosquitoes [167]. A recent study on *An. gambiae* has reported a novel role of cytoplasmic actin as an extracellular pathogen recognition factor that mediates phagocytosis of bacteria and serves as an antagonist in *Plasmodium* infection. *An. gambiae* actin interacts with specific extracellular immune factors and binds to the bacterial surfaces, mediating their phagocytosis [168].

### 2.4. RNAi

RNAi is a sequence-specific RNA-mediated silencing process. Noncoding RNAs are involved in silencing genes in a specific manner. RNAi has evolved as a primitive immune response triggered by the presence of foreign nucleic acid and has been reported in both vertebrates and invertebrates, including algae, plants, and fungi, but not bacteria [169]. It was first identified as an antiviral defense mechanism in plants [170]. In insect systems, a pathogenic infection triggers this phenomenon as the first level of defense. Evidence found in the last few years suggests that RNAi is a vital antiviral as well as anti-transposon defense system in insects [171]. In case of viruses of human health importance such as RNA viruses, studies have shown that viral genome-derived DNA causes persistent infection in their insect host [172,173].

Noncoding RNAs have been classified into three major classes on the basis of secondary structure, length, template from which they are derived, processing, and the mechanism of action: miRNA (~18–24 nt), siRNA (~18–24 nt), and piRNA (~24–30 nt). Almost all the noncoding RNAs are processed from dsRNA sequences through dicer activity. These small RNAs are loaded onto a multiprotein complex, called RNA-induced silencing complex (RISC), which guides the recognition of target RNA through a specific protein member, Argonaute which leads to the silencing of genes. Each of these classes is discussed in detail in the following sections.

#### 2.4.1. siRNA Pathway

Viral infection primarily activates the siRNA pathway in mosquitoes. Traditionally, siRNAs are produced by the cleavage and processing of double-stranded RNA (of exogenous or endogenous origin) and lead to binding with complementary sequences in mRNA, causing the degradation of mRNA. Depending upon the origin of the substrate, siRNA has two distinct roles: it is involved in regulating cellular processes such as heterochromatin formation [174] and chromosomal segregation [175] through the binding of endogenously encoded dsRNA, or it is involved in antiviral immunity by the processing of the exogenous RNA originated from viruses (or viRNA) [171]. The pathway is triggered by Dicer2, R2D2, and Ago2, with orthologs present in almost all mosquito groups [176].

- *Biogenesis of siRNA*

**Figure 3:**
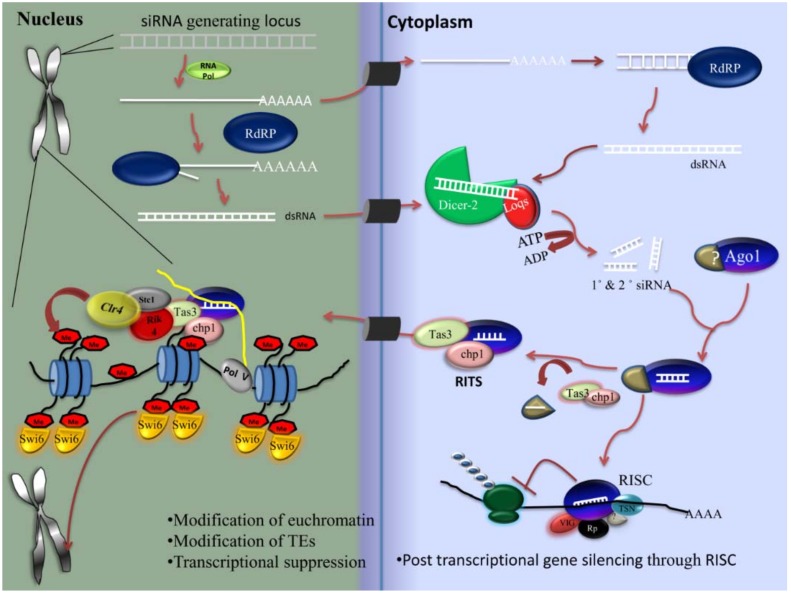
Endogenous siRNA processing and role of these siRNAs in nucleus and cytoplasm. In the nucleus, siRNA-generating locus produces ssRNA, which is converted into dsRNA by RNA-dependent RNA polymerase in the nucleus and cytoplasm. In cytoplasm, this dsRNA is processed by Dcr2 and Loqs complex leading to the formation of endogenous duplex siRNA population (1° & 2° siRNAs duplex form), which is further converted into siRNA by the action of Ago1 and loaded to the RNA-induuced transcriptional silencing (RITS) complex. RITS is a multiprotein complex containing chromodomain protein chp1, argonaute interacting protein tas3. This complex is transported into the nucleus and binds with a nascent transcript in a sequence-specific manner, which leads to the recruitment of Stc1 and Rik4 proteins (the CLRC complex). This complex further recruits the Clr4, that is, methyltransferase, and attaches the methyl group on H3K9 (9th position of lysine in histone-3). H3K9 methylation stabilizes Swi6 (HP1 protein), which leads to the formation of heterochromatin or the silencing of transposons. In cytoplasm, the RISC complex is involved in post-transcriptional gene silencing.

Exogenous dsRNAs are long, linear, perfectly base-paired double-stranded RNAs taken up as foreign nucleic acid into the cell and introduced directly into the cytoplasm for processing. These dsRNAs are recognized by Dicer through its RNA-binding domain, which contains endonuclease catalytic activity (RNase III-type). The Dicer complex cleaves dsRNA into siRNA duplexes, which are ~21–28 nucleotide long moieties, contain 3′ hydroxyl termini, and 2-nucleotide 3′ overhangs with 5′ phosphate, which are recognized by the PAZ domain of Dicer. Meyers et al. showed that apart from the exogenous origin of siRNAs, siRNAs originate endogenously from genes located in the pericentromeric, transposable element and repetitive regionws of the host genome [177] (Figure 3)**.** RNA polymerase IV and RNA polymerase V (RNA Pol V) play a role in the biosynthesis of endogenous siRNA. It has also been reported that RNA Pol V is necessary for siRNA-mediated DNA methylation [178]. The major function of RNA Pol V is to methylate DNA or histone protein at siRNA-generating loci and promote siRNA formation in an indirect manner, as methylation on histone or DNA gives the feed-forward loop for the production of siRNAs. RNA Pol V also generates noncoding transcripts with the help of DRD1 (chromatin remodeling protein) and DMS3 (structural maintenance of chromosomal hinge domain protein), which is further involved in heterochromatin formation mediated by siRNA [179].

- *Components of the siRNA pathway*

The molecular mechanisms of siRNA revolve around three major components, namely, Dicer, Argonaute, and R2D2. Dicer is a member of the ribonuclease III family and is a key factor for the biosynthesis of most of the small RNA molecules. It cleaves long, double-stranded RNA into small RNAs that act as regulatory RNA. Dicer contains a phosphate-binding site, which identifies the phosphorylated 5′ end of small RNAs [180]. The RNase domain of Dicer is responsible for the cleavage of one strand of the dsRNA [181]. During a viral infection, Dicer is also involved in the processing of viral dsRNAs/RNA intermediates, which ultimately produce viral siRNAs [182]. The Argonaute (Ago) protein family plays a central role in the RNAi mechanism by binding with small RNAs, leading to the silencing of complementary transcripts in the cell by either degrading them or inhibiting their translation. The Ago family possesses several homologs across vertebrates and invertebrates. In insects, Ago2 is involved in siRNA-based antiviral immunity [183]. Another important molecule in the siRNA pathway is R2D2, which was first identified as a partner of the Dicer protein in *Drosophila*. In small RNA biosynthesis, it has both a positive and a negative role. R2D2 is involved in the transmission of the siRNA guide strand from Dicer-2 to Ago2 and inhibits the processing of miRNA precursors by Dicer-2 [184] and prevents endo-siRNA sorting into Argonaute-1 [185]. R2D2 acts as a bridge between the initiation and the effector steps of the RNAi pathway by promoting the passage of siRNA from Dicer to RISC [186].

- *Mechanism*

**Figure 4:**
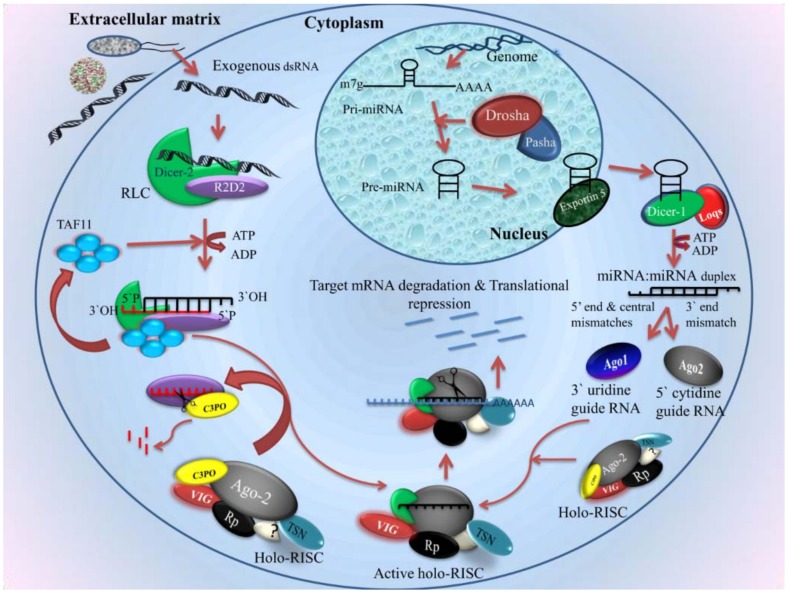
The processing and mechanism of action of exogenous siRNA and miRNA. In the nucleus, pri-miRNA is produced by a genome which is processed by Drosha and Pasha and converted into Pre-miRNA. Pre-miRNA is transported to the cytoplasm through exportin 5, which is further processed by Dicer-1 and the Loqs complex in the cytoplasm. A miRNA–miRNA* duplex is formed, which is involved in the formation of guide RNA by the degradation of passenger strand. Loading of the miRNA–miRNA* duplex to argonaute is sequence-specific. The 5′ end and central mismatch in the miRNA–miRNA* duplex are sorted into Argonaute 1, which leads to the formation of 3′ uridine guide RNA. The 3′end mismatch in the miRNA–miRNA* duplex is sorted into Argonaute 2, which leads to the formation of 5′ cytidine guide RNA. Exogenous dsRNA from different sources are processed in the cytoplasm by RLC complex (Dcr2 and R2D2) into small RNA (sRNA) duplex. The activity of the RLC complex is enhanced by TAF11. Holo-RISC complex is made up of different proteins: Ago2 or Ago1, VIG, Dicer-2 or Dicer-1, TSN, ribosomal proteins, and other unknown proteins. Incorporation of the sRNA duplex into Ago2 leads to the formation of active RISC complex and the passenger strand is degraded by C3PO, an endonuclease. In case of both miRNA and siRNA, the active RISC complex containing guide strand binds to target mRNA, leading to its translational suppression or degradation of target mRNA.

The biogenesis of siRNA occurs in the cytoplasm in a stepwise manner [187]. This process involves the association of Dicer with specific members of Argonaute proteins and other effector proteins, leading to the formation of RISC (Figure 4). First, Dicer cleaves the exogenous and endogenous long dsRNA into ~21–25 nucleotide siRNA in the duplex form. This duplex contains 2-nucleotides 3′ overhanging with 3′-OH and 5′ phosphate termini [188]. This siRNA duplex is formed by a pre-RISC complex with the Dicer protein [189] and hsc70/hsp90 chaperone system [190]. In *Drosophila*, the RISC loading complex is produced by R2D2, Dicer2, and TATA-binding protein-associated factor 11 (TAF11) [191]. TAF11 is a tetrameric protein that localizes in both the cytoplasm and the nucleus. In the nucleus, it acts as a nuclear transcriptional factor that is involved in cell development and viability [191]. In the cytoplasm, it stabilizes the tetrameric complex of RLC formed by R2D2 and Dicer-2 and promotes the activity of RLC. TAF11, Dcr-2, and R2D2 are present in particular cytosolic foci called D2 bodies. Liang et al. reported a 10-fold increase in the binding of siRNA over tetrameric RLCs that contain TAF11, suggesting that TAF11 enhanced RLC activity. The incorporation of the siRNA duplex into Argonaute-2 leads to the formation of the RISC. Ago2 eliminates one of the strands, known as the passenger strand, and retains the other strand, known as the guide or active strand [191]. Selection of the guide strand is dependent upon the thermodynamic stabilities of the 5′ end of small RNA duplex [192]. C3PO is a Mg^2+^-dependent endoribonuclease enzyme. This enzyme is involved in the activation of the RISC through the removal of cleavage products of the passenger strand [193]. The activated RISC contains the guide RNA and Argonaute-2, which degrades mRNAs containing perfectly complementary sequences [194]. The PIWI domains of Ago2 contain an aspartate–aspartate–glutamate motif [195]. This motif is involved in the degradation of mRNA through the RNase-H enzyme-like activity [196]. The fragments of mRNA are further degraded by the distinct cytosolic enzymes [197].

#### 2.4.2. Micro RNA in Mosquito Immunity

miRNAs are a small class of noncoding RNAs 19–24 nucleotides in length, regulating gene expression by degrading mRNA or halting transcription by binding to the 3′ untranslated region (UTR) of a gene [127]. miRNAs are initially expressed as primary miRNAs by RNA pol II in the nucleus after being derived from introns of protein-coding genes or exons of noncoding genes [198] (Figure 4). The stem–loop structure present in the primary miRNA is later cleaved by Drosha, a ribonuclease III enzyme, in association with another accessory protein, Pasha, which results in the production of precursor miRNA [199]. In cases where Drosha-mediated cleavage is not involved, the miRNAs are called mirtrons [200,201]. Further, Exportin-5 helps the precursor miRNA to be transported into the cytoplasm, followed by terminal loop excision by Dicer-1 along with loquacious protein, to create an miRNA duplex. In this miRNA duplex, the passenger strand usually gets degraded and the guide miRNA gets incorporated into the RISC, which then interacts with its target to affect its expression.

miRNAs are involved in many cellular functions, ranging from the development to providing immunity during infections. Each miRNA can regulate the expression of several cellular mRNAs [202]. Depending upon the miRNA binding to their target regions, whether complementarity is partial or complete, expression of their target is inhibited either by suppression of translation or by degradation of mRNA [203]. Studies have shown that miRNA binding to their targets may also result in the induction of expression of genes in some cases [204]. miRNAs isolated from different species of insects, including mosquitoes, are regularly being deposited to the miRBase database [205].

In the case of viral infection in insects, double-stranded viral replication intermediates trigger RNAi as a defense [206]. Silencing of Dicer and Argonaute genes through dsRNA was shown to lead to an increase in ONVV titers, signifying the role of RNAi as an important antiviral defense [207]. In addition, various viral suppressors of RNAi have been reported for insect viruses [208,209]. As siRNA components are shown to be involved in a number of antiviral responses compared to miRNAs, it shows a higher rate of sequence variations [210]. Additionally, it was shown that a gene *Ars2*, is involved in the miRNA pathway, where it interacts with a microprocessor complex to stabilize pri-miRNA and siRNA pathways involving Dicer-2 and Ars2 interaction, which promotes the target cleavage. Insect cells of *Drosophila* were more susceptible to RNA viruses where the *Ars2* gene was silenced, further showing the importance of this protein in insect RNAi [211].

In RNAi-mediated insect immunity, miRNAs play an important role in controlling pathogens. Studies have shown that not necessarily pathogens but even a simple blood meal can induce immunity-related genes in a mosquito [212]. Nevertheless, they play an important role during infection. In a study, aga-miR-305 has been shown to increase the susceptibility of *An. gambiae* for *Plasmodium falciparum*, which serves as an example of a parasite hijacking the cellular repertoire of miRNAs for its own replication [213]. Not just the malaria parasite, but RNA viruses such as Zika virus, dengue virus, and chikungunya virus, as well as bacterial infection with *Wolbachia*, also elicit an immune response in mosquitoes [214,215,216,217]. The role of miRNAs in mosquito immunity can be executed by controlling the expression of immune-related genes and genes destined to bring post-translational modifications or by directly binding to the genome of the pathogen. Among the viruses that infect mosquitoes, the Zika virus also triggers the mosquito host RNAi response, where replicative dsRNA intermediate is a target of Dicer2 [216]. Additionally, miR-252 has been shown to act as an antiviral for DENV-2 by regulating the expression of the E protein of DENV-2 in the case of *Ae. albopictus* [218].

#### 2.4.3. PIWI Pathway

**Figure 5:**
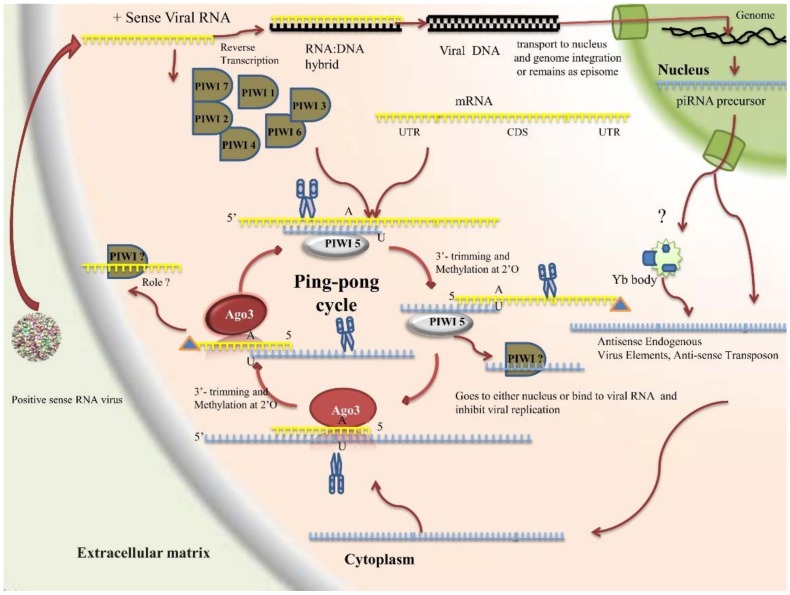
PIWI pathway in mosquitoes. Upon RNA virus infection, as the RNA strand reaches the cytoplasm of the host, it is either utilized by PIWI protein or the viral genome integrates into the host genome after reverse transcription, replication, and integration. In the first category, as viral RNA genome enters the host cells, it is bound with unknown PIWI protein (PIWI 1-7) and is directly fed into the ping-pong cycle, thereby amplifying the piRNAs. RNA from viral genome or mRNA binds to complementary antisense RNA bound to Piwi5. Viral RNA or mRNA is cleaved at 5′ end and trimmed at 3′ end by nucleases and methylated at 2′-OH. Antisense RNA is then released bounded to an unknown PIWI protein and binds to viral RNA inhibiting viral replication, whereas processed sense RNA binds to Ago3 and to precursor long-length antisense piRNA. Antisense precursor is then cleaved at 5′ end and 3′ end by nucleases and methylated at 2′-OH. The sense piRNA is released although its function is not known, whereas antisense piRNA binds to Piwi5 and starts another round of ping-pong cycle. In the second category, the viral RNA genome is reverse transcribed and replicated, which then enters into the host cell nucleus and then integrates into the genome. It is later transcribed and transported to the cytoplasm, and fed into ping-pong cycle, although it is not known whether it directly binds to complementary RNA bound to Ago3 or there is a role of Yb body as in the case of germinal cells.

The PIWI pathway, best known for its role in protecting germ cells from transposon elements, was first discovered in *D. melanogaster* and later reported in other organisms [219]. This is the least understood pathway of RNAi and is proposed to play a role in germline development and maintenance [220], providing a protective mechanism against transposable elements in epigenetic programming [220,221] and having a crucial role during viral infection ka [222]. They range 21–32 nucleotides in length (centered around 27 nucleotides in *Aedes* [223] and 25 in *Drosophila* [224]), are processed from long-length single-stranded RNA, are associated with the PIWI class of Argonaute proteins, and form piRISC (Figure 5). Mutation in the PIWI protein leads to infertility because of the increased transposon activity, whereas knockdown causes the loss of body regeneration and the failure in body maintenance in Planaria [225]. Most studies on the PIWI pathway have been performed on *Drosophila*; however, a few studies carried out on *Aedes* reveal that the mechanism differs in a few aspects from that of *Drosophila*; for instance: (1) the presence of piRNA, proved in both somatic and germ cells in *Aedes*, as well as majorly in germ cells in the case of *Drosophila*; (2) absence of the Aub gene in *Aedes*, with its role being performed by Piwi5; and (3) the lack of antiviral activity in the piRNA pathway of *Drosophila*, whereas the *Aedes* piRNA system appears to have antiviral activity, as evidenced in the case of infection of *Aedes* with the Semliki forest virus [226].

- *Source of piRNA*

Initially, it was thought that piRNAs originated from transposable elements. However, analysis of various insect species revealed a huge disparity in the levels of transposable elements present in them. For instance, nearly half of the *Aedes* genome, ~17% of *An. gambiae*, and 24% of *Culex* genome codes for transposable elements [227]. However, according to the TEfam database, only 24% piRNA maps to transposons. Studies have now shown that piRNAs are generated from cellular noncoding (2%) or protein-coding 3′ UTRs (8%) (for example, Histone gene) and from unannotated regions (40%). The annotated genes were found to consist of the sequence of RNA virus (Rhabdovirus) and nonretroviral sequences (NIRV), which were found to be integrated into *Aedes* genome and were of antisense orientation. It has also been proved that antisense piRNA of the *Histone* gene regulates the cell cycle by down-regulating the mRNA level of the *Histone* gene [228]. Primary transcripts were transcribed by RNA Pol II from defective transposable elements, and the pathway seems to be devoid of Dicer protein, the crucial component of siRNA and miRNA pathways.

In *Ae. albopictus*, it has been found that most of the sense piRNA are derived from short interspersed nuclear elements whereas most of the antisense piRNAs were derived from DNA transposons. Codon sequences (CDS)-derived piRNA dominates the 5′ UTR and 3′ UTR piRNAs in all stages of life. In CDS-derived piRNAs, the sense piRNAs comprise of half or more than half of the total, whereas, in 5′ UTR and 3′ UTR, antisense piRNA dominates. It has been observed that piRNA may be generated upon certain physiological conditions such as blood feeding. Several novel piRNAs have been identified upon blood feeding in *Aedes* [227]. Viral genomes are another source of piRNAs and can be generated from different sources within the genome. Upon infection with alphaviruses in *Aedes*, vpiRNA are generated from subgenomic RNA that is transcribed from the internal promoter (5′ side of the capsid gene); in the case of Flaviviruses, however, the piRNAs are generated from the distinct region. Even in *Aedes*, different viruses are processed by different members of the PIWI pathway; for example, Sindbis virus RNA is processed in a ping-pong manner via Piwi5 and Ago3, whereas dengue RNA can also be processed by Piwi6 [229]. In addition, viral RNA is transcribed into vDNA, which remains as an episome or it may integrate into the genome and may serve as a source of vpiRNAs [229].

- *Components*

*Aedes* codes for eight PIWI proteins (Piwi1–7 and Ago3). Of these, Piwi5 and Ago3 form the core of the viral piRNA ping-pong amplification loop, whereas other proteins are involved in transposon-derived piRNA biogenesis [228]. Additionally, the components show a high level of divergence between different mosquito species. In the case of *Aedes*, there are four homologs of Piwi4, three homologs of Piwi5, and two homologs of Armitage, whereas, in the case of *Culex*, there are three homologs of Piwi4 and Piwi5 each and two homologs of Armitage. There is only one homolog for all these proteins in *Drosophila* and *An. gambiae*, and there is only one copy of Ago3 and Spn-E in all [230].

- *piRNA characteristics and generation mechanism*

piRNAs arise from repetitive genomic sequences, also called piRNA clusters, ranging in size from 20–100 kb, in a Dicer-independent manner [226,227]. They are antisense in orientation to transposon, causing silencing by binding to complementary sequences. Nascent transcripts arising from piRNA clusters are loaded onto the 5′ end of the PIWI protein. The PIWI protein also shows base preferences in their binding to piRNA. Piwi5 prefers to bind to antisense having uridine at its 5′ end, whereas sense piRNA binds to Ago3-associated proteins with ‘A’ in its 10th position [231]. Furthermore, the 3′ end is methylated at 2′-OH, which is recognized by the PAZ domain of PIWI proteins [223]. Studies have elucidated that there is an enrichment of sense and antisense piRNA of the *Histone* genes in Aag2 cells during infection with Sindbis virus [228]. piRNAs are first transcribed from piRNA clusters as ssRNA by RNA pol II and then transported to the cytoplasm with the help of RNA-binding proteins. Once in the cytoplasm, the precursor piRNA interacts with helicases, which, in turn, interacts with the endonuclease. After processing the piRNA with 5′ uridine (specificity of primary piRNA), they bind to PIWI proteins, and this PIWI–piRNA intermediate interacts with the exonuclease that caused 3′ end processing, facilitated by the Tudor protein. The length of piRNA is determined by the associated PIWI proteins [232]. These are then cleaved and loaded onto the complex for further processing and then modified at the 3′ end (2-*O*-methylated) in the cytoplasm, making them more stable. Processed piRNA are then transported back to the nucleus [225]. As an alternate mechanism, selective amplification of piRNA is carried out in a secondary pathway, also called the ping-pong cycle, which targets transposon elements. The piRNA from the primary pathway interacts with the PIWI pathway protein. This PIWI protein/piRNA complex binds to the transposon and cleaves it between the 10th and the 11th position of piRNA. The 5′ end of the cleaved transposon is degraded, and the 3′ end is bound to the PIWI proteins. The RNA is enriched in adenine, and it is complementary to the uridine of the piRNA. The RNA is further processed similarly to primary piRNA [233]. Recently it has been established that the Piwi4 protein interacts with Dicer-2 and Ago2 of the exo-siRNA pathway and Piwi5 and Piwi6 of the PIWI pathway, directly or indirectly, thus, connecting the siRNA and the piRNA pathways [226].

- *Roles*

In addition to their widely known roles, piRNAs were found to be responsible for the resistance of host against a particular virus, as seen in the endogenous Bornavirus-like nucleoprotein elements, which were reverse-transcribed and integrated into primate and rodent genomes, causing resistance to bornaviral infection [228]. It has also been found that some arboviruses are transmitted vertically, suggesting that the PIWI pathway may have a role in the prevention of vertical transmission via germ cell [234]. During infection, viral nucleic acids occasionally integrate into the host genome. They are called endogenous viral elements (EVE) or nonretroviral integrated RNA virus sequences (NIRVS). In the case of RNA viruses infection (CHIKV, DENV, WNV, and so forth), a portion of the genome is reverse-transcribed into the vDNA episome and then inserted into the genome. Computational analysis of the mosquito genome has shown that Flavivirus and Rhabdovirus are the sources of NIRVS in mosquitoes, whereas other virus-derived elements were either rare or absent. These elements were found in *Ae. aegypti* and *Ae. albopictus* and rarely in *Anopheles* or *Culex* [235]. It is assumed that during infection with a particular virus, the NIRVS elements of the virus bind to the complementary sequence and then provide immunity to the host [236].

#### 2.4.4. Viral Counter Defenses to the RNAi Pathway

As explained in the earlier sections, RNAi is used as a weapon by hosts ranging from plant to mammals and insects to defend against pathogens, including viruses, halting the pathogen load. To counteract the host’s defense, viruses have evolved strategies for employing proteins that target the RNAi components. A study demonstrated that alphavirus infection induces high RNAi response in mosquitoes, which led to the generation of viral-derived small RNA (viRNA). Suppression of these viRNAs by the over-expression of viral protein resulted in increased viral replication, affecting the mortality rate of mosquitoes [237]. These viral proteins are called viral suppressors of RNAi (VSRs) [238]. These proteins are known to co-evolve with the host in a bid to maintain a balance between both host growth and pathogen. Generally it has been found that proteins showing VSR activity are either enzymes (protease, targeting proteins of RNAi pathway, RNases which degrade RNA) or RNA-binding proteins (binds to long RNA and prevents its slicing by dicer or binds to small RNA and prevents its interaction with RNAi pathway proteins) [239,240,241]. However, in some other instances, capsid can also possess RNAi suppressor activity [242]. Samuel reported that the capsid protein of Yellow fever virus binds to dsRNA and inhibits the dicer activity. The presence of basic amino acids (in the case of RNA-binding proteins) and GW/WG motif (Ago2 interaction) is common in VSRs [171]. The most commonly known targets of VSRs in RNAi are Dicer-2 (Flock House virus and *Drosophila* C virus), Ago2 (Cricket paralysis virus and Nora virus), and RNA (Nodamura virus, *Culex* Y virus, and *Drosophila* X virus) [243]. VSRs from different viruses having the same target are found to have very less similarity in sequence but have a similar structure, indicating their common ancestor—for example, the B2 protein of Wuhan Noda Virus and between Nodamura Virus and Flock House Virus—is less than 30% homology [171]. The B2 protein binds to double-stranded RNAs and inhibits the formation of siRNA [244]. Whereas other viral suppressors protein acts through sequestration of siRNA including p19 protein of tombusviruses and p21 protein of Beet yellow viruses [245]. The P38 capsid protein of turnip crinkle virus binds to long dsRNAs and duplex siRNA structures and mediates VSR activity. It also interacts with the argonaute-1 protein of Arabidopsis [246,247].

## 3. Future Perspectives

This review reveals the underlying mechanism of innate immunity of mosquitoes against pathogens. Studying the immune system of mosquitoes will provide insights into significant opportunities to link tissue damage, immune invasions mechanisms, and immune response against pathogens. More importantly, unraveling the riddles of mosquito’s immune system will shed light on the fight against disease-spreading pathogens. Understanding the triggers that allow pathogens to grow and replicate in mosquitoes and those that restrict the pathogens to survive in low levels will provide insights into the mechanisms of mosquito-pathogen interactions. Finding the exact immune evasion strategies of pathogens will help to create effective ways to control them. Although successful pathogen dissemination in the mosquito midgut, hemocytes, fat body, salivary glands, and all tissues depends on tissue barriers such as MIB, MEB, SGIB, and SGEB, there is a lack of knowledge about the exact mechanisms and barriers at the molecular and biochemical levels. The know-how of molecules involved in signaling, tissue damage, activation, and regulation of the immune response, recognition of pathogens, and signaling pathways that activate the immune system of mosquitoes is essential for a better control of the host–pathogen interactions. Several methodologies based on molecular imaging, new molecular technologies, and biochemical and genomic analyses will strengthen the studies of immune mechanisms and provide insights into the basis of the functionality of each component that can further be utilized to evaluate host-pathogen interactions.

## Supplementary Materials

The following are available online at http://www.mdpi.com/2075-4450/9/3/95/s1, Figure S1: PRISMA 2009 flow diagram.
