# Supplementary material for: Mosquito Innate Immunity
Source: Insects. 2018 Aug 8;9(3):95. doi: 10.3390/insects9030095 (PMC6165528; doi:10.3390/insects9030095)
Supplement: Supplementary file 1 [file insects-09-00095-s001.pdf]

Supplementary figure 1: PRISMA 2009 Flow Diagram

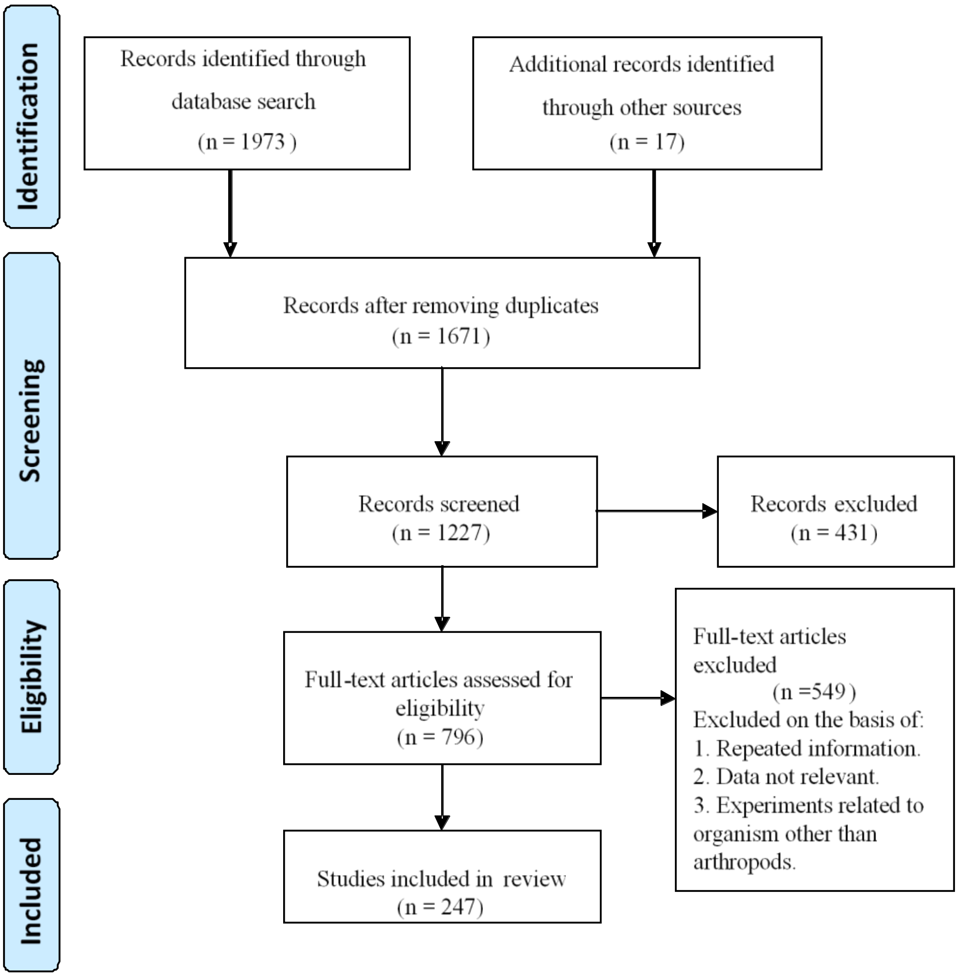

From: Moher D, Liberati A, Tetzlaff J, Altman DG, The PRISMA Group (2009). *P*referred *R*eporting *I*tems for *S*ystematic Reviews and *M*eta- *A*nalyses: The PRISMA Statement. PLoS Med 6(7): e1000097. doi:10.1371/journal.pmed1000097

For more information, visit www.prisma-statement.org.
